# Molecular Landscape and Validation of New Genomic Classification in 2668 Adult AML Patients: Real Life Data from the PETHEMA Registry

**DOI:** 10.3390/cancers15020438

**Published:** 2023-01-10

**Authors:** Claudia Sargas, Rosa Ayala, María José Larráyoz, María Carmen Chillón, Estrella Carrillo-Cruz, Cristina Bilbao-Sieyro, Esther Prados de la Torre, David Martínez-Cuadrón, Rebeca Rodríguez-Veiga, Blanca Boluda, Cristina Gil, Teresa Bernal, Juan Miguel Bergua, Lorenzo Algarra, Mar Tormo, Pilar Martínez-Sánchez, Elena Soria, Josefina Serrano, Juan Manuel Alonso-Domínguez, Raimundo García-Boyero, María Luz Amigo, Pilar Herrera-Puente, María José Sayas, Esperanza Lavilla-Rubira, Joaquín Martínez-López, María José Calasanz, Ramón García-Sanz, José Antonio Pérez-Simón, María Teresa Gómez-Casares, Joaquín Sánchez-García, Eva Barragán, Pau Montesinos, on behalf of PETHEMA group

**Affiliations:** 1 Grupo Acreditado de Investigación en Hematología, Instituto de Investigación Sanitaria La Fe (IIS La Fe), 46026 Valencia, Spain; 2 Hospital Universitario 12 de Octubre, National Cancer Research Center, Complutense University, 28041 Madrid, Spain; 3 CIMA LAB Diagnostics, Departamento de Bioquímica y Genética, Universidad de Navarra, 31008 Pamplona, Spain; 4 Servicio de Hematología, Hospital Universitario de Salamanca (HUS/IBSAL), CIBERONC, Centro de Investigación del Cáncer–IBMCC (USAL–CSIC), 37007 Salamanca, Spain; 5 Hospital Universitario Virgen del Rocío, Instituto de Biomedicina (IBIS/CSIC/CIBERONC), Universidad de Sevilla, 41013 Sevilla, Spain; 6 Hospital Universitario de Gran Canaria Dr. Negrín, 35010 Las Palmas de Gran Canaria, Spain; 7 Instituto Maimónides de Investigación Biomédica de Córdoba (IMIBIC), Hospital Universitario Reina Sofía, Universidad de Córdoba (UCO), 14004 Córdoba, Spain; 8 Servicio de Hematología, Grupo Acreditado de Investigación en Hematología, Hospital Universitario y Politécnico La Fe, Instituto de Investigación Sanitaria La Fe (IIS La Fe), 46026 Valencia, Spain; 9 Centro de Investigación Biomédica en Red de Cáncer (CIBERONC), Instituto de Investigación Sanitaria La Fe (IIS La Fe), 46026 Valencia, Spain; 10 Hospital General Universitario de Alicante, 03010 Alicante, Spain; 11 Hospital Universitario Central de Asturias, Instituto Universitario (IUOPA), Instituto de Investigación del Principado de Asturias (ISPA), 33011 Oviedo, Spain; 12 Hospital Universitario San Pedro de Alcántara, 10003 Cáceres, Spain; 13 Hospital Universitario General de Albacete, 02006 Albacete, Spain; 14 Hospital Clínico Universitario–INCLIVA, 46010 Valencia, Spain; 15 Hospital Universitario Fundación Jiménez Díaz, 28040 Madrid, Spain; 16 Hospital Universitario General de Castellón, 12004 Castellón de la Plana, Spain; 17 Hospital Universitario Morales Messeguer, 30008 Murcia, Spain; 18 Hospital Universitario Ramón y Cajal, 28034 Madrid, Spain; 19 Hospital Universitari Dr. Peset, 46017 Valencia, Spain; 20 Complexo Hospitalario Lucus Augusti, 27003 Lugo, Spain; 21 Servicio Análisis Clínicos, Grupo Acreditado de Investigación en Hematología, Hospital Universitario y Politécnico La Fe, Instituto de Investigación Sanitaria La Fe (IIS La Fe), 46026 Valencia, Spain

**Keywords:** acute myeloid leukemia, Next–Generation Sequencing, cross–validations, mutational profile, genomic classification, clinical validation

## Abstract

**Simple Summary:**

Next–Generation Sequencing (NGS) has provided a deeper genetic understanding of acute myeloid leukemia (AML) that has been recently incorporated into AML classification and risk–stratification guidelines. Single molecular analysis has become inefficient and molecular testing based on NGS is emerging as an irreplaceable diagnostic tool in clinical settings. The PETHEMA cooperative group has constituted a nationwide NGS network with centralized analysis in seven high–skilled laboratories. The study of molecular profiles in the “real–life” PETHEMA cohort supports the increasing role of NGS on the clinical management of AML patients.

**Abstract:**

Next–Generation Sequencing (NGS) implementation to perform accurate diagnosis in acute myeloid leukemia (AML) represents a major challenge for molecular laboratories in terms of specialization, standardization, costs and logistical support. In this context, the PETHEMA cooperative group has established the first nationwide diagnostic network of seven reference laboratories to provide standardized NGS studies for AML patients. Cross–validation (CV) rounds are regularly performed to ensure the quality of NGS studies and to keep updated clinically relevant genes recommended for NGS study. The molecular characterization of 2856 samples (1631 derived from the NGS–AML project; NCT03311815) with standardized NGS of consensus genes (*ABL1*, *ASXL1*, *BRAF*, *CALR*, *CBL*, *CEBPA*, *CSF3R*, *DNMT3A*, *ETV6*, *EZH2*, *FLT3*, *GATA2*, *HRAS*, *IDH1*, *IDH2*, *JAK2*, *KIT*, *KRAS*, *MPL*, *NPM1*, *NRAS*, *PTPN11*, *RUNX1*, *SETBP1*, *SF3B1*, *SRSF2*, *TET2*, *TP53*, *U2AF1* and *WT1*) showed 97% of patients having at least one mutation. The mutational profile was highly variable according to moment of disease, age and sex, and several co–occurring and exclusion relations were detected. Molecular testing based on NGS allowed accurate diagnosis and reliable prognosis stratification of 954 AML patients according to new genomic classification proposed by Tazi et al. Novel molecular subgroups, such as mutated *WT1* and mutations in at least two myelodysplasia–related genes, have been associated with an adverse prognosis in our cohort. In this way, the PETHEMA cooperative group efficiently provides an extensive molecular characterization for AML diagnosis and risk stratification, ensuring technical quality and equity in access to NGS studies.

## 1. Introduction

Introduction of Next–Generation Sequencing (NGS) into routine molecular diagnosis has provided deep molecular knowledge of acute myeloid leukemia (AML). These findings have allowed for the refinement of classification and risk stratification systems based on recurrent genetic abnormalities.

In 2016, Papaemmanuil et al. proposed the first genomic classification of AML that identifies 11 molecular classes, each with distinct diagnostic features and clinical outcomes [1]. This classification has been recently revised and updated in Tazi et al., 2022, proposing 16 molecular classes based on cytogenetics and the mutational status of 32 genes [2]. The importance of genomic characterization has also been reflected in the recently revised World Health Organization (WHO) Classification [3], new International Consensus Classification (ICC) [4] and European LeukemiaNet (ELN) risk stratification [5], which prioritize genetic abnormalities to establish diagnosis and prognosis to evaluate measurable residual disease (MRD) and to select treatment.

In this situation, molecular analysis by single–gene techniques has become inefficient in order to provide a complete characterization of AML. In contrast, NGS represents a more sensitive tool to capture all the relevant molecular markers in one assay and is widely recommended to study the molecular landscape of this disease [6].

NGS implementation to perform accurate diagnosis in AML is currently demanded by physicians and patients. However, introduction of NGS into clinical routine faces novel challenges [7]. NGS requires large batches of samples in order to be cost–effective, workflows are time–consuming, and interpretation needs highly qualified specialists. Moreover, the diversity of NGS panels, platforms and quality control criteria might prevent the success of the approach [8]. Hence, to efficiently introduce NGS into routine molecular diagnostics, it is necessary to establish quality requirements and to standardize gene panels and variant reporting.

In order to provide comprehensive NGS studies to AML patients and to guarantee equity of access, the PETHEMA cooperative group (Programa Español de Tratamientos en Hematología) has established a nationwide network of central laboratories aimed to harmonize NGS results under consensual criteria in newly diagnosed and relapsed/refractory AML patients [9].

This study summarized the NGS–AML project (NCT03311815), reporting quality control assays and the molecular profile of 2668 AML patients reported in the PETHEMA AML registry. We show co–occurring and mutual exclusion relationships among genes and distinct molecular profiles according to disease stage, age and sex and genomic classification in the “real–life PETHEMA cohort”.

## 2. Materials and Methods

### 2.1. Development of the Diagnostic Platform

Implementing NGS studies in the routine molecular diagnosis of AML patients requires specialization, budgetary stability and logistical support. The PETHEMA cooperative group established a nationwide network of NGS studies for fast and standardized molecular diagnosis of AML. This strategy aims to provide coverage of NGS studies to 38.5 million inhabitants distributed in geographical areas ranging from 2.2 to 8.9 million habitants. For this purpose, seven centers with logistical and technical capacity for the management of a high number of samples were designed as reference laboratories for the centralization of samples submitted by PETHEMA institutions in each area. In this way, a large territory and population was covered. The platform was supported by PETHEMA in logistical management, as well as, closer and well–established relationships between the sample referral institution and the assigned central laboratory. The designated reference centers for NGS analysis concentrate a large number of AML samples, allowing for the rapid completion of the sequencing runs and their management by highly specialized staff.

### 2.2. Study Design and Reference Laboratories

We show a prospective, multi–center, non–interventional and translational biomedical research, performed in seven Spanish PETHEMA reference laboratories: Hospital Universitario La Fe (HULF, Valencia, Spain), Hospital Universitario de Salamanca (HUS, Salamanca, Spain), Hospital Universitario 12 de Octubre (H12O, Madrid, Spain), Hospital Universitario Virgen del Rocío (HUVR, Sevilla, Spain), Hospital Universitario Reina Sofía (HURS, Córdoba, Spain), Hospital Universitario de Gran Canaria Dr. Negrín (HUDN, Las Palmas de Gran Canaria, Spain), CIMA LAB Diagnostics (UNAV, Pamplona, Spain).

### 2.3. Consensus Genes Establishment

The development of the diagnostic platform required several meetings to coordinate criteria on which genes should be analyzed based on their clinical relevance in AML. After extensive bibliographic revision of current molecular basis of AML, all reference laboratories should assess by NGS the mutational status of genes that define the diagnosis and prognosis as well as guide treatment options (*ASXL1*, *BCOR*, *CEBPA*, *EZH2*, *FLT3*, *IDH1*, *IDH2*, *NPM1*, *RUNX1*, *SF3B1*, *SRSF2*, *STAG2*, *U2AF1*, *ZRSR2* and *TP53*). Moreover, there was a recommendation for the study of other genes with proven evidence on their relevance in AML pathogenesis (*ABL1, BRAF, CALR, CBL, CSF3R, DNMT3A, ETV6, GATA2, HRAS, JAK2, KIT, KRAS, MPL, NRAS, PTPN11, SETBP1, TET2* and *WT1*).

The establishment of consensus genes has enabled laboratories to work with the NGS platforms and panels according to their individual requirements, which have largely enabled the development and maintenance of the diagnostic platform. NGS panels and platforms used by each center are described in Supplementary Materials Tables S1 and S2.

### 2.4. NGS Standardization Procedures and Cross–Validation Rounds

The diagnostic platform established a quality control assay by exchanging control samples among reference laboratories every 9–12 months. To date, three cross validation (CV) rounds have been performed. As previously reported, in the first and second CV rounds, minimum quality parameters [uniformity (>85%) and mean read depth of 1000X] and consensus recommendations for variant report [All centers should report: (1) mutations in relevant genes for clinical guidelines, targeted therapy and risk stratification and (2) all pathogenic variants detected with VAF >5% excepting those described at hotspot regions which will be reported up to 1% VAF] were stablished. For both CV rounds, 10 samples harboring 54 variants were distributed. In first CV round, VAF for all variants was >5% while in the second CV round, 5 from 30 total variants had low VAF ranging from 1.8–4.9%. In the third CV round, to explore the accuracy and reliability of low VAF variants (<5%) detection, 4 samples harboring 32 variants (11 VAF < 5%) were analyzed, according to previously established criteria [9].

### 2.5. Patients and Inclusion Criteria

All adult patients (≥18 years) with newly diagnosed or relapsed/refractory AML (excluding acute promyelocytic leukemia) according to the World Health Organization criteria (2008 and 2016), regardless of the treatment received, were eligible for mutational profile study by NGS. The Institutional Ethics Committee for Clinical Research of each institution approved this study. Written informed consent in accordance with the recommendations of the Declaration of Human Rights, the Conference of Helsinki, and institutional regulations were obtained from all patients.

### 2.6. Clinical Validation

Clinical validation was performed based on the new genomic classification which proposes unified framework for disease classification and risk–stratification in AML based on cytogenetic analysis and an NGS–panel of 32 genes [2]. Molecular class defining genes were: ***NPM1***, ***TP53***, ***WT1***, ***CEBPA***, ***DNMT3A***, ***IDH1***, ***IDH2***, *ZRSR2*, ***U2AF1***, ***SRSF2***, ***SF3B1***, ***ASXL1***, *STAG2*, *BCOR*, ***RUNX1***, ***EZH2***, *MLL*, *PHF6*, *SF1*, *NF1*, *CUX1*, ***SETBP1***, ***FLT3*** and ***TET2***. * Bold genes represent PETHEMA consensus genes.

This new classification categorizes AML in 16 molecular classes with different prognostic values and encompass the established WHO entities: “WHO2016 set 1” [inv(16), t(8;21) and *NPM1*] and “WHO2016 set 2” [t(11;x), t(6;9), inv(3) and *CEBPA*bi]; and also novel categories: “*TP53* and complex karyotype (CK)”, “sAML1” (Mutated *SRSF2*, *SF3B1*, *U2AF1*, *ASXL1*, *EZH2*, *RUNX1* or *SETBP1*), “sAML2” (More than one mutations in sAML1 genes including *DNMT3A* and *TET2*), “*WT1*”, “Trisomies”, “*DNMT3A* + *IDH1/2*”, “Not class defining mutations (mNOS)” and “No events” category. Since our study excludes acute promyelocytic leukemia, category “t(15;17)” is not applicable.

This classification also proposes an integrated risk score based on the 16 molecular classes: “*NPM1*”, “inv(16)”, “t(8;21)”, “*CEBPA*bi” and “No events” define the favorable risk group; “sAML1”, “t(6;9)”, “*WT1*”, “mNOS”, “t(11;X)”, “*DNMT3A*–*IDH1/2*” and “trisomies” the intermediate and “*TP53*–CK”, “sAML2” and “inv(3)” the adverse risk group. Genomic groups and sub–classifications are summarized in Supplementary Table S3. Internal tandem duplications (ITD) in *FLT3* are the only genetic alterations with independent prognosis value from class membership. These mutations were not considered as “class defining” alterations as they are represented in all classes but modulate risk groups classification as follows: In the favorable risk group, patients with mutated *NPM1* who also harbored a *FLT3*–ITD mutation were reclassified to the intermediate risk group. Similarly, a *FLT3*–ITD mutation reclassifies intermediate risk patients to an adverse risk group. Moreover, in order to assess the prognostic impact of *TP53* configurations, we classified patients according *TP53* mono–allelic or multi–hit as described by Tazi et al.: Mono–allelic: One *TP53* mutation with VAF ≤ 65%; and multi–hit: Two *TP53* mutations or one *TP53* mutation with VAF > 65% or one *TP53* mutation + del(17).

### 2.7. Statistics

All statistics were performed using SPSS version 22 (IBM, Armonk, NY, USA) and GraphPad Prism 4 (GraphPad, La Jolla, CA, USA) software programs. Chi square test was used to assess associations between categorical variables. Survival analyses were performed using the Kaplan–Meier method and the log–rank test. The Cox proportional–hazards model was used to evaluate the risk of death among groups. *p*–value (*p*) < 0.05 was considered as a statistically significant test.

## 3. Results

### 3.1. Third Cross Validation Round

In the third CV round, the error rate (ER) for variants with VAF > 5% decreased from previous rounds: 1st: 39%, 2nd: 14.4% and 3rd: 4.76%. However, the ER in variants with VAF < 5% increased from 28.6% (five variants with mean VAF 3.3%) to 59.6% (11 variants with mean VAF 1.2%). ER, mean VAF and standard deviation (SD) for the last CV round are summarized in Supplementary Table S4 and Figure S1. Therefore, the diagnostic platform maintained: (1) the cut–off of VAF > 5% to report clinically relevant variants and (2) the criteria to report only low VAF variants (<5%) in hotspot regions with strong clinical evidence, suggesting variant confirmation in an additional sample.

### 3.2. Baseline Demographics and Molecular Profile in NGS–AML Protocol Cohort

From October 2017 to October 2019, NGS analyses were performed in 1631 samples from 1471 AML patients enrolled in the NGS–AML protocol (NCT03311815), with available clinical date (i.e., treatment approach). Disease status at samples collection was: 1268 diagnosis (DX), 204 relapse (REL) and 159 refractoriness (RES) (Table 1).

An additional cohort of 1225 samples analyzed by NGS between November 2019–May 2021 has been included only for molecular characterization of AML: 1166 (DX), 47 (REL) and 12 (RES). Only those samples that met NGS quality requirements established by the diagnostic platform were included in the study. Overall, 2856 samples were analyzed (2434 DX, 251 REL, and 171 RES).

### 3.3. Summary Mutation Profile

In the global cohort (N = 2856 samples), 7768 variants were detected. A total of 96.5% of samples showed at least 1 mutation, mean 2.7 mutations/sample (range 0–9). Most patients had three variants (21.1%), followed by patients with two (20.9%) and four (17.8%). *FLT3* (24.6%), *DNMT3A* (24.3%), *NPM1* (22.4%) and *TET2* (21.6%) were the most frequently mutated genes (Supplementary Figure S2). According to ELN–2022 [5], 85.3% of patients at diagnosis showed at least one mutation in clinically relevant genes to establish the diagnosis, prognosis or to select treatment.

### 3.4. Co–Mutations and Exclusivity Patterns

*NPM1*, *FLT3* and *DNMT3A* were significantly co–mutated for all combinations (*p* < 0.001). *PTPN11* and *NPM1* showed a strong association (*p* < 0.001) as well as *PTPN11*–*DNMT3A* (*p* = 0.0017) and *PTPN11*–*FLT3* (*p* = 0.024). Mutations in *NPM1* were highly associated with mutated *IDH1* (*p* < 0.001) but were exclusive with R172–*IDH2* (*p* < 0.001). In contrast, *DNMT3A* mutations were highly associated with both mutated *IDH1* and *IDH2* (*p* < 0.001). *CEBPA* was frequently co–mutated with *GATA2* (*p* < 0.001); and *ASXL1*, *RUNX1* and *SRSF2* were strongly associated with each other (*p* < 0.001).

**Figure 1:**
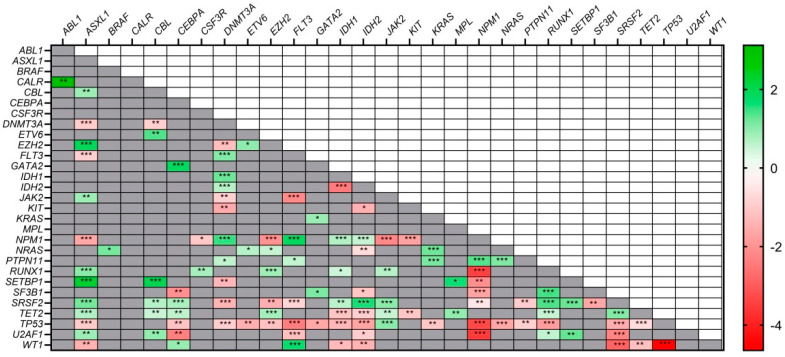
Co–occurrence and mutual exclusivity patterns among genes. Red: exclusive relationship; Green: co–occurring relationship. Higher color intensity indicates stronger association: * *p* < 0.05, ** *p* < 0.01, *** *p* < 0.001.

On the other hand, mutations in *TP53* were the most exclusive with all the analyzed genes (*p* < 0.05). Mutations in *IDH1* and *IDH2* were also mutually exclusive of each other (*p* < 0.001). Mutated *NPM1* was highly exclusive with mutations in: *RUNX1* (*p* < 0.001), *SRSF2* (*p* < 0.001) and *ASXL1* (*p* < 0.001). The main association and exclusivity patterns for all genes are shown in Figure 1.

**Figure 2:**
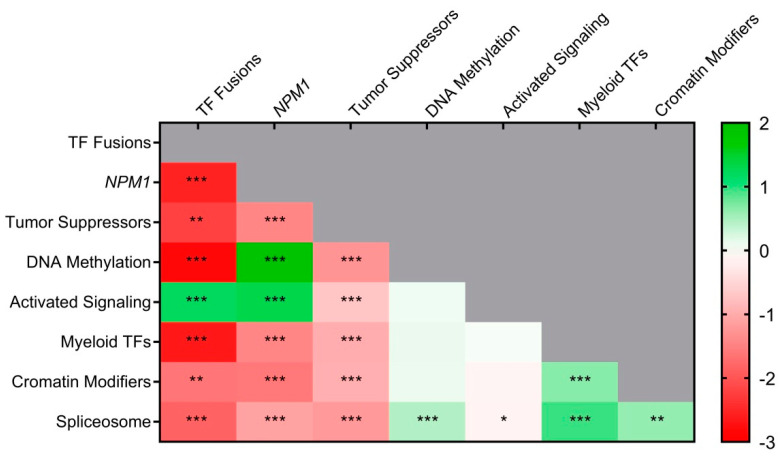
Heatmap of association and exclusivity patterns among functional categories. Red: exclusivity relationship; Green: co–occurring relationship. Higher color intensity indicates stronger association. * *p* < 0.05, ** *p* < 0.01, *** *p* < 0.001.

We also identified co–occurring and exclusivity patterns according to functional categories of AML [10]. Genes were grouped into nine categories based on their biological function (Supplementary Table S5). We identified commonly co–occurring events between “transcription–factor (TF) fusions” and “activating signaling genes” (*p* < 0.001). Associations were also found between “*NPM1*” with “DNA–methylation genes” (*p* < 0.001) and “activating signaling genes” (*p* < 0.001)” as well as co–occurring events between “Spliceosome–genes” with “myeloid TFs” (*p* < 0.001), “chromatin modifiers” (*p* < 0.01) and “DNA–methylation genes” (*p* < 0.001). On the other hand, several mutually exclusive relationships were observed: “*NPM1*” mutations were highly exclusive with “myeloid TFs” (*p* < 0.001), “Spliceosome–genes” (*p* < 0.001), and “chromatin–modifying” (*p* < 0.001); mutations in “Spliceosome–genes” were highly exclusive with “TF fusions” (*p* < 0.001) and remarkably, the “Tumor suppressor–genes” category was highly exclusive with all other functional categories (*p* < 0.001) (Figure 2).

### 3.5. Disease Stage Mutational Profile

**Figure 3:**
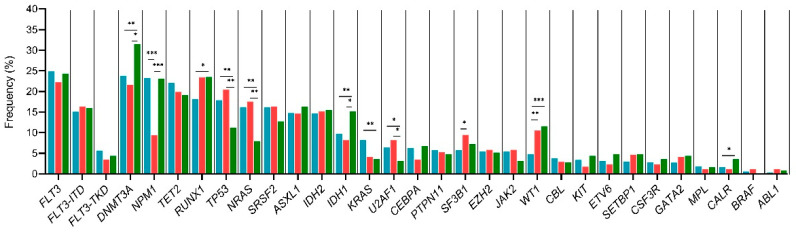
Mutational frequency according to disease stage. Blue bars: Diagnosis, green bars: Relapse and red bars: refractoriness. * *p* < 0.05, ** *p* < 0.01, *** *p* < 0.001. ITD: internal tandem duplication; TKD: tyrosine kinase domain.

Subgroup analyses based on disease stage (DX or REL or RES) showed statistically significant results: *NPM1* (*p* < 0.001) and signaling pathway genes such as *KRAS* (*p* = 0.007) and *NRAS* (*p* < 0.001) were more frequently mutated at diagnosis. In refractory AML, *WT1* (*p* < 0.001) was more frequent, meanwhile relapse AML exhibited more mutations in *RUNX1* (*p* = 0.037), *DNMT3A* (*p* = 0.018), *IDH1* (*p* = 0.017) and *WT1* (*p* < 0.001). Mutational frequency and *p*–values for each correlation are described in Supplementary Table S6A, Figure 3.

### 3.6. Age–Related Mutational Profile

**Figure 4:**
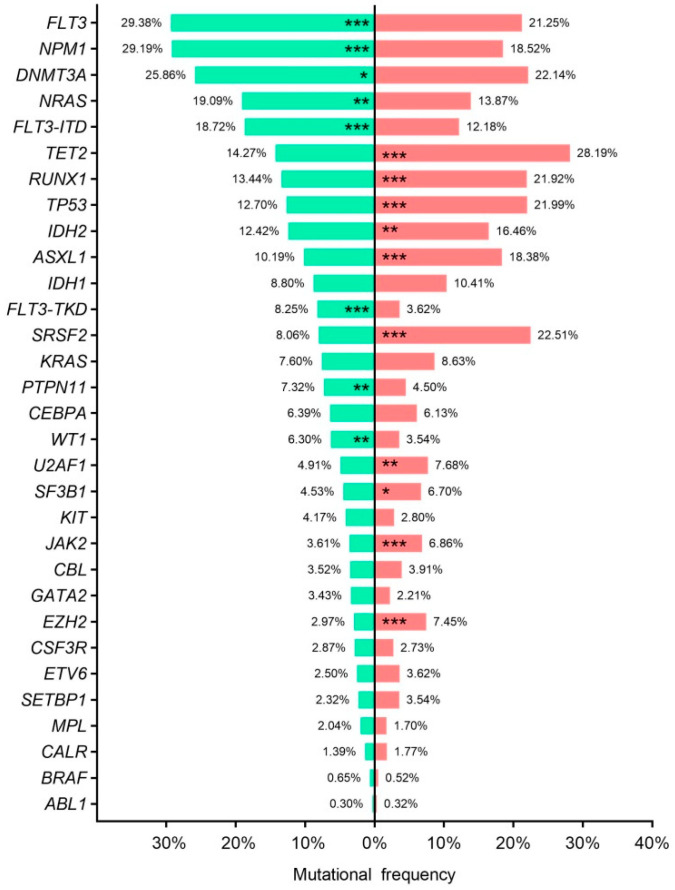
Age–related mutational profile. Bar chart representing mutational frequencies according to age at diagnosis. Green; <65 years old, red; ≥65 years old. * *p* < 0.05, ** *p* < 0.01, *** *p* < 0.001. ITD: internal tandem duplication; TKD: tyrosine kinase domain.

NGS studies revealed distinct mutational profile in young (<65 years–old) and elderly (≥65 years–old) AML patients. The mean number of gene mutations at diagnosis was higher in older patients than younger (2.9 ± 0.04 vs. 2.5 ± 0.04; *p* < 0.001). Older patients also had a higher frequency of *TET2* (*p* < 0.001), *RUNX1* (*p* < 0.001), *TP53* (*p* < 0.001), *IDH2* (*p* < 0.01), *ASXL1* (*p* < 0.001), *SRSF2* (*p* < 0.001), *U2AF1* (*p* < 0.01), *SF3B1* (*p* = 0.028), *JAK2* (*p* < 0.001) and *EZH2* (*p* < 0.001). In contrast *FLT3* (*p* < 0.001), *NPM1* (*p* < 0.001), *DNMT3A* (*p* = 0.032), *NRAS* (*p* < 0.01), *PTNP11* (*p* < 0.001) and *WT1* (*p* < 0.001), were frequently mutated in young AML. Mutational frequencies and *p*–values are shown in Supplementary Table S6B, Figure 4.

### 3.7. Sex–Related Mutational Profile

**Figure 5:**
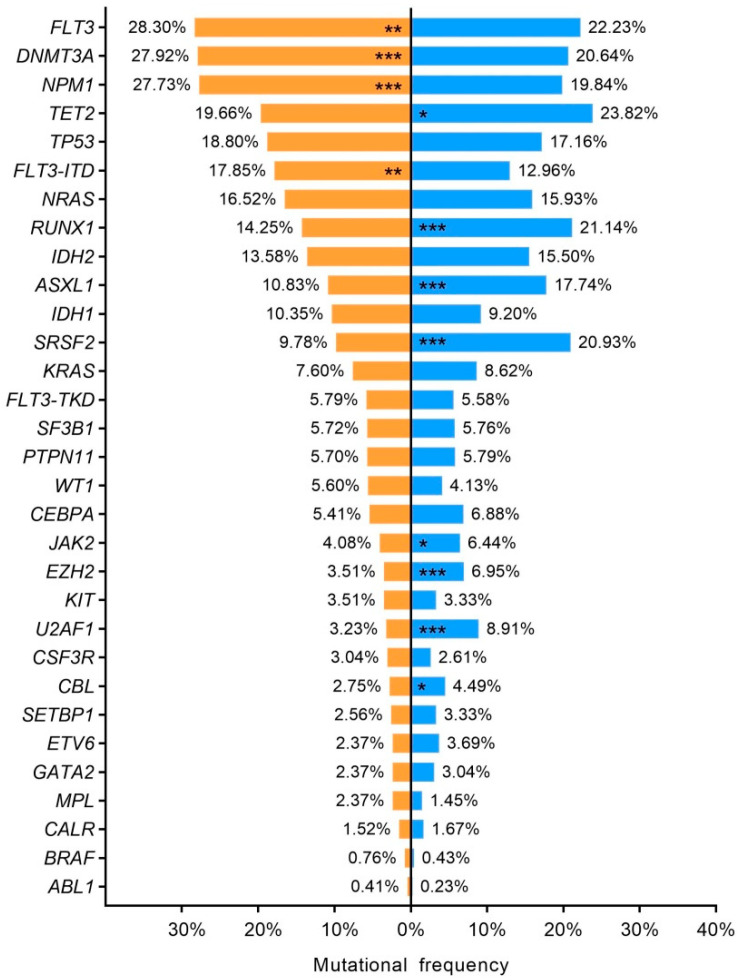
Sex–related mutational profile. Bar chart representing mutational frequencies. Orange; female, blue; male. * *p* < 0.05, ** *p* < 0.01, *** *p* < 0.001. ITD: internal tandem duplication; TKD: tyrosine kinase domain.

Our cohort showed a well–balanced distribution between male (56.2%) and female (43.8%) patients, similar to that described in previous AML cohorts [1,10]. Sex–specific mutational profiles were observed. Females harbored lower number of mutations than male patients (2.8 ± 0.04 vs. 2.6 ± 0.04; *p* < 0.01). Mutations in *DNMT3A* (*p* < 0.001), *FLT3* (*p* < 0.01) and *NPM1* (*p* < 0.001) were overrepresented in females and *TET2* (*p* = 0.014), *RUNX1* (*p* < 0.001), *ASXL1* (*p* < 0.001), *SRSF2* (*p* < 0.001), *EZH2* (*p* < 0.001), *U2AF1* (*p* < 0.001), *JAK2* (*p* = 0.01) and *CBL* (*p* = 0.025) mutations in male patients (Supplementary Table S6C, Figure 5).

### 3.8. Paired Samples and Mutation Stability

Molecular profiling of paired samples was conducted in 97 patients at diagnosis–relapse (DX–REL) and 59 patients at diagnosis–refractoriness (DX–RES). In DX–REL comparison, loss events were more frequently observed than gain events (43.7% vs. 26.4%) in relapse samples. Interestingly, 17.2% of patients showed simultaneous mutation loss and gain events, while 24.1% maintain the diagnosis’ mutational profile. The most stable mutated genes were *TP53*, *WT1* and *NPM1*, with stability rates of 81.3%, 80% and 77.8%, respectively. In contrast, signaling activating genes were found to be highly unstable: *KIT*, *FLT3*–ITD and *FLT3*–TKD mutations, *NRAS*, *KRAS* and *PTPN11* showed stability rates below 50%. Moreover, while mutations in *KIT*, *FLT3*–TKD, *KRAS* and *PTPN11* were almost equally lost and acquired, mutations in *NRAS* and *FLT3*–ITD were predominantly lost (Supplementary Figure S3).

In refractory AML, 49.2% of patients retained the mutational status found at diagnosis. *NPM1* mutations were also the most stable gene at refractory AML (83.3%). Signaling activating genes were highly unstable: *NRAS*, *KRAS*, *PTPN11* and *FLT3*–TKD showed stability rates below 45%, being more frequent the loss of these mutations (Supplementary Figure S4).

### 3.9. New Genomic Classification Applied to PETHEMA–NGS Cohort

**Figure 6:**
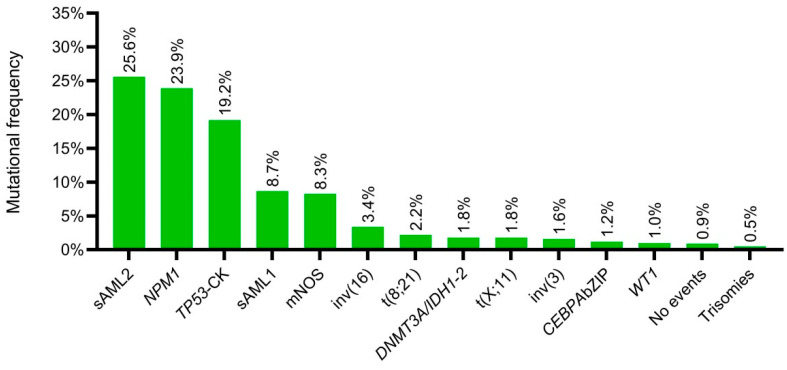
Molecular classes’ distribution according to the Tazi et al., 2022 genomic classification. CK: Complex karytotype. mNOS: Not class defining mutations.

Based on the availability of clinical, cytogenetic and mutational data, 954 patients were eligible to assess the updated genomic classification by Tazi et al. [2]. The most frequently mutated classes were: “sAML2” (25.4%), “*NPM1*” (23.9%), and “*TP53*–CK” (19.2%). Other less frequent classes were: “sAML1” (8.7%) and “Not class defining mutations” (8.3%). Molecular classes’ distribution is shown in Figure 6. The “*CEBPA*bi” category was underrepresented in the PETHEMA cohort (0.8%). Therefore, based on novel diagnosis and prognosis classifications of AML, patients with in–frame mutations in basic leucine zipper domain of *CEBPA* (*CEBPA* bZIP) were assessed as a biological AML subgroup (1.2%). In this regard, WHO entities [inv(16), t(8;21), *NPM1*, *CEBPA* bZIP, t(11;x), t(6;9) and inv(3)] represented the 34% of PETHEMA–cohort.

#### 3.9.1. Prognosis Value of Molecular Classes

Molecular classes have also been associated with different prognostic values. The previously established WHO categories, “inv(16)” (Median OS not reached at 42 months), “*CEBPA* bZIP” (Median OS not reached at 32 months) and “*NPM1*” [29.0 months (95%CI 19.9–38.0)] had the best outcomes while inv(3) had the worst prognostic value [4.9 months (95%CI 0.8–9.1)]. Among new molecular classes, those patients without driver mutations “No events” (Median OS not reached at 33 months) or “Not class defining mutations” [23.3 months (95%CI 11.0–35.6)] showed the best prognostic value while “*TP53*/CK” [5.3 months (95%CI 2.9–7.6)], “sAML2” [12.1 months (95%CI 9.9–14.2)] and *WT1* [4.0 months (95%CI 0.0–18.4)] classes had the worst OS (*p* < 0.001) (Table 2; Supplementary Figure S5). When we evaluated the prognostic value according to the mono–allelic (N = 47; 32.9%) or multi–hit (N = 96; 67.1%) status of *TP53* mutations, we did not find a different outcome between both groups [mono–allelic: 5.4 months (95%CI 0.007–10.7); multi–hit: 4.1 months (95%CI 2.9–5.3) (*p* = 0.088)] (Supplementary Figure S6).

**Figure 7:**
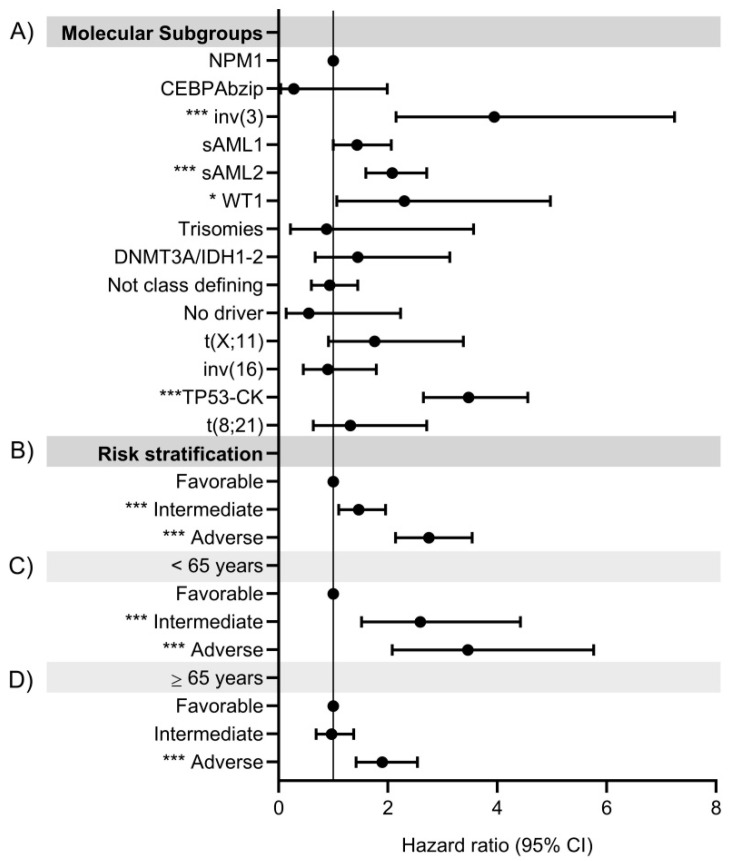
Hazard ratio for death according to (**A**) New molecular subgroups in the global cohort; and new genomic risk score for: (**B**) Global cohort, (**C**) Patients < 65 years and (**D**) Patients ≥65 years. * *p* < 0.05, *** *p* < 0.001.

In terms of risk of death, the molecular classes “inv(3)” [3.9 (95%CI 2.1–7.2) (*p* < 0.001)] and “*TP53*/CK” [3.5 (95%CI 2.6–4.6) (*p* < 0.001)] showed the highest risk of death compared to “*NPM1*” class. Remarkably, the new established “sAML2” [2.1 (95%CI 1.6–2.7) (*p* < 0.001)] and “*WT1*” [2.3 (95%CI 1.1–5) (*p* < 0.05)] categories were the next with higher risk of death (Figure 7A, Supplementary Table S7A).

#### 3.9.2. Integrated Risk Score

We also assessed the integrated risk score based on cytogenetic and gene mutations proposed in the new genomic classification [2]. In the evaluable cohort (N = 954), 23.8% of patients were classified to a favorable risk group, 27.1% were included in the intermediate risk group and 49.1% in the adverse risk group. We also evaluated the impact of age at diagnosis in the new genomic classification. Risk stratification distribution was significantly different between age groups: <65 years (intensive = 416; non–intensive = 10) vs. ≥65 years (intensive = 150; non–intensive = 378). Young patients showed homogeneous distribution of the different risk groups (Favorable: 31.2%, Intermediate: 32.4% and adverse: 36.4%), while older patients were predominantly included in the adverse risk group (Favorable: 17.8%, Intermediate: 22.9% and adverse: 59.3%; *p* < 0.001).

**Figure 8:**
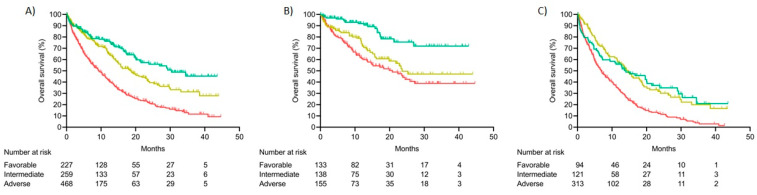
Overall survival according to new genomic risk score: (**A**) Global cohort, (**B**) Patients < 65 years, (**C**) Patients ≥ 65 years.

In terms of outcomes, median OS was 30.8 months (95%CI NR) in the favorable risk group, 18.5 months (95%CI 14.3–22.7) in the intermediate group and 9.4 months (95%CI 7.8–11.00) in the adverse risk group (*p* < 0.001) (Figure 8A). Intermediate and adverse risk patients had 1.5 (95%CI 1.1–2.0; *p* < 0.01) and 2.7 (95%CI 2.1–3.5; *p* < 0.001) increased risk of death relative to favorable risk group (Figure 7B, Supplementary Table S7B).

We also found differences in terms of OS in both age groups. Median OS in favorable–risk group was not reached at 42.8 months in patients < 65 years, while intermediate and adverse risk groups reached a median OS of 23.6 (95%CI NR) months and 20.8 (95%CI 13.2–28.3), respectively (*p* < 0.001) (Figure 8B). In contrast, median OS of older patients was significantly decreased for all risk groups: Favorable (14.0; 95%CI 6.3–21.8), intermediate (14.6; 95%CI 11.9–17.4) and adverse (6.9; 95%CI 5.6–8.3) (*p* < 0.001) (Figure 8C).

Regarding risk score, young patients classified in the intermediate and adverse risk group showed 2.6 (95%CI 1.5–4.4; *p* < 0.001) and 3.5 (95%CI 2.1–5.8; *p* < 0.001) higher risk of death relative to those patients classified in the favorable risk group (Figure 7C, Supplementary Table S7C). On the other hand, patients >65 years classified in the adverse risk group showed an increased risk of death of 1.9 (95%CI 1.4–2.5; *p* < 0.001) compared to those classified as favorable risk. No statistically significant results were found in the risk of death of intermediate risk patients: 1.0 (95%CI 0.7–1.4; *p* = 0.864) (Figure 7D, Supplementary Table S7D). In general, patients >65 years had a dismal prognosis and higher risk of death compared to younger ones for all risk groups: Favorable: 4.7 (95%CI 2.8–8.0; *p* < 0.001), intermediate: 1.8 (95%CI 1.2–2.6; *p* < 0.01) and adverse: 2.6 (95%CI 2.0–3.5; *p* < 0.001).

##### Comparison between the Integrated Risk Score and 2022 ELN Risk Classification

**Figure 9:**
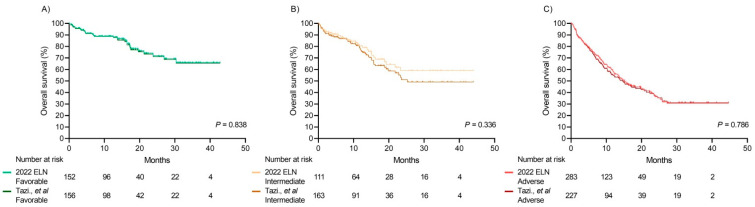
Overall survival curves according to 2022 ELN risk classification and Tazi et al. [2] genomic AML classification: (**A**) Favorable, (**B**) Intermediate and (**C**) Adverse risk groups.

According to current 2022 ELN risk stratification, we selected 546 fit patients for a tentative comparison with the AML genomic classification risk score. We did not find a distinct OS (2022 ELN vs. Tazi et al.) for favorable (Median OS not reached in both groups; *p* = 0.839), intermediate (Median OS not reached vs. 25.3 months; *p* = 0.336) or adverse–risk patients (Median OS 15.2 months vs. 14.7 months; *p* = 0.786) according to both classifications (Figure 9).

## 4. Discussion

NGS has become the preferred technology to capture the heterogeneous molecular landscape of AML [2,3,4,5]. These approaches have been rapidly adopted as a potential routine tool for molecular diagnosis in AML patients [11]. However, its translation into clinical practice is hampered by specific requirements, such as the necessity of highly skilled laboratories, the increased cost compared to single–gene assays and the expected high turnaround time [7,12]. In this scenario, our results show that an NGS diagnostic platform, established by the PETHEMA cooperative group, was able to provide NGS reliable results with all relevant molecular data currently required for diagnosis and prognosis stratification and therapy choice. In addition, this kind of cooperative study allows for the assessment of the genetic heterogeneity of AML in large cohorts of patients and provides an extensive quality molecular data to evaluate current genomic knowledge in “real–life” AML cohorts.

Due to the rapid NGS implementation into routine molecular diagnosis of AML, NGS workflows and quality specifications are heavily reliant on laboratory specific procedures [13,14]. Therefore, current NGS analyses are characterized by the lack of standardized procedures, the diversity of quality metrics criteria, and the high variability of the assessed genes and variant reporting criteria [15]. To address the need for harmonization procedures, the PETHEMA cooperative group implemented the first Spanish nationwide NGS testing strategy. Regular rounds of cross–validation were planned in order to identify weaknesses and to establish consensus quality metrics criteria for variant reporting among seven central laboratories in AML molecular diagnosis.

Networking for NGS studies allowed us to identify challenges in its clinical implementation [9]. The first CV round revealed the absence of AML key genes in some NGS approaches, and consequently a consensus set of relevant genes for the clinical management was defined. Next steps of the diagnostic platform enabled us to unify variant reporting according to the role of genes in diagnostic classification, prognostic utility and targeted therapy. Cooperative studies have also allowed us to address technical challenges. Comparison of results between centers facilitates discrimination of polymorphic variants and sequencing artifacts from real AML–related variants [7].

Our results demonstrated that NGS standardization in the context of a cooperative group is possible with a concordance of 96.6% in variant detection (VAF > 5%). Noteworthy, the detection of low VAF (<5%) variants (concordance 41.2%) was also consistent with previous studies, which report that accurate detection of low VAF variants by NGS could be compromised. CV rounds results reflected the improvement of the diagnostic platform performance as the error rate decreased in variants VAF > 5% as a result of the experience gained in NGS studies. Although the second CV round included 5 variants with a mean VAF of 3.3%, in the third CV round we aim to explore the performance of NGS studies in very low VAF variants. For this purpose, we included 11 variants with a mean VAF of 1.2%. Data analysis revealed an increase of the ER between both CV rounds due to the very low VAF variants of the third CV round. Consequently, we established VAF 5% cut–off for variant reporting, although according to other specific studies, variants in hotspot regions were reported up to 1% VAF even at borderline technical quality [16,17,18].

Our results reflect that a comprehensive NGS approach is suitable for defining the molecular profile of AML as over 96% of patients of the “real–life” PETHEMA cohort harbored at least one mutated gene. Our study also reflects the genomic heterogeneity that encompasses AML (13,14) as several gene–to–gene interactions but also co–occurrence and mutual exclusivity patterns across functional categories were described. Recently, published studies have highlighted the impact of co–mutations in modulating treatment response [19,20].

Similarly, distinct mutational profiles were detected among disease stages, reflecting the clonal evolution of AML [1,21]. In fact, when compared to the diagnosis’ molecular profile we detected fewer mutational changes in resistance to induction therapy (51%) than relapse AML patients (87%) which may suggest different mechanisms underlying both moments [22,23,24,25]. Clonal evolution of the disease could be especially relevant for treatment response [26,27], and in relapsed AML patients, our results supported that molecular testing should be conducted again in order to identify targetable abnormalities such as *FLT3* mutations, which showed a stability rate of 43.3% in our cohort [28].

In addition to mutational changes according to disease stages, specific mutational profiles have been associated to patient’s clinical characteristics, such as sex and age, with significant impact on prognosis, therapeutic allocation and disease monitoring [29]. In fact, we report that young patients show a mutational profile very similar to female AML patients, which allows them to benefit more from targeted therapies due to higher frequency of *FLT3* mutations [30,31,32,33]. In contrast, male and elderly patients show a molecular profile with a higher frequency of adverse risk genetic abnormalities and limited targeted therapy opportunities [34,35,36]. Indeed, some studies suggest that these features should be considered as an essential variable in clinical trials to deepen understanding of the disease and to identify new treatment opportunities [37,38].

Molecular diagnosis of AML has shifted towards a comprehensive mutational study driven by NGS, yielding large amounts of data. Updated diagnostic (WHO and ICC) and prognostic (ELN) classifications [3,4,5] include an increasing number of genetic abnormalities, which may challenge its applicability in many countries and institutions who cannot afford molecular data integration. However, comprehensive molecular analyses, as the recent revision of the genomic classification of AML are needed to understand the clinical relevance of molecular biomarkers to define novel clusters with prognostic value. In our comparison between genomic AML classification and 2022 ELN risk stratification we did not find differences in OS for favorable, intermediate and adverse risk groups. However, for a comprehensive comparison it would be necessary to evaluate the prognosis impact of individual genetic abnormalities in larger cohorts. Mutations in sAML genes are considered in both risk classification proposals with different prognostic impact [2,5]. Although ELN guidelines associate mutations in “myelodisplasia–related genes” as adverse risk regardless of the number of mutations, our results found significant differences in the OS between sAML1 and sAML2 patients, as described by Tazi et al., (*p* = 0.018). In this sense, further studies are needed in order to certainly clarify the impact of sAML mutations on prognosis.

Regarding *TP53* mutations, in the PETHEMA cohort, 47 patients were included in the mono–allelic group (32.9%), while 96 were included in the multi–hit group (67.1%). Similar results were described in the Tazi et al. cohort: mono–allelic (31.1%) vs. multi–hit: (68.9%). We did not find a distinct outcome between these groups, although the sample size was limited to draw solid conclusions. Our results are concordant with Tazi et al., who concluded that the allelic state of *TP53* (mono allelic or multi–hit) provide no further prognostic information in AML.

On the other hand, recent studies reported that instead of biallelic mutations, only in–frame mutations in the bZIP domain of *CEBPA* should be considered as a favorable prognosis marker [39]. In our cohort, the percentage of patients with *CEBPA* bZIP mutations is similar to the *CEBPA*bi subgroup reported by Tazi et al., (1.2% vs. 1.8%) and in both cases, OS analysis includes them within a favorable risk subgroup. However, *CEBPA*bi and *CEBPA* bZIP may be overlapping categories in some patients as several studies suggest that *CEBPA* double mutated frequently includes mutations in the C–terminal region which allocated bZIP domain [40]. Moreover, we believe that the recent recognition of in–frame mutations in the bZIP domain of *CEBPA* as a prognostic biomarker may allow for more homogeneous analysis by reducing the variability in the interpretation of the biallelic character of these mutations [41].

## 5. Conclusions

This report reflects the efforts of the PETHEMA cooperative scientific group to adopt a nationwide strategy network of reliable and consistent NGS analyses. The establishment of consensus subset genes and the periodic CV rounds have strengthened the diagnostic network by unifying analysis criteria and decreasing reporting variability. Molecular analyses through NGS are routinely performed for AML patients and a comprehensive molecular profile of the disease is offered to clinicians in order to individualize the therapeutic strategy. Moreover, NGS results have provided a large amount of molecular data that has revealed the molecular complexity of the disease. In this cohort, mutual exclusion and mutational co–occurrences among genes and functional categories have been deciphered and a distinct molecular profile between age groups at diagnosis and sex has been detected. Moreover, clinical validation of the current genomic classification in the “real–life” PETHEMA cohort has demonstrated the correlation of the molecular subgroups with clinical prognosis, reflecting the utility of the cooperative NGS studies in routine molecular diagnostics.

## Figures and Tables

**Table 1:** Diagnosis cohort (N = 1268). Demographic and baseline characteristics.

Characteristic	Mean	Median	Range	N	(%)
**Age, years**	64.9	67.7	18–98	1268	100
<65				540	42.6
≥65				728	57.4
**Sex**				1268	100
Male				712	56.2
Female				556	43.8
**ECOG**				1075	100
0				420	39.1
1				452	42.0
2				135	12.6
3				53	4.9
4				15	1.4
Not available				193	
**WBC (×10^9^/L)**	32.8	8.8	0.24–407	1118	
**BM blast cells, %**	53.4	52.0	0–100	1026	
**Creatinine, mg/dL**	1.1	0.90	0.28–10.3	1071	
**MRC cytogenetic profile**				1011	100
Favorable				57	5.6
Intermediate				178	17.6
Unfavorable				269	26.6
Normal karyotype				507	50.1
Not available				257	
**AML FAB subtype**				715	100
M0				88	12.3
M1				144	20.1
M2				126	17.6
M4				173	24.2
M5				144	20.1
M6				31	4.3
M7				9	1.3
Not available				553	
**Therapeutic approach**				1268	100
Intensive				695	54.8
Non–intensive				513	40.5
Supportive care only				60	4.7
**Type of AML**				1268	100
De novo				920	72.6
Secondary				348	27.4

**Table 2:** Median overall survival and 95% CI for molecular AML classes.

Molecular Classes	OS	(95% CI)		*p*
		Lower IC	Upper IC	
inv(16)	NR			<0.001
*CEBPA* bZIP	NR			
No events	NR			
*NPM1*	29.0	19.9	38.0	
Not class defining mutations	23.3	11.0	35.6	
*DNMT3A/IDH1–2*	18.5	1.7	35.3	
sAML1	18.1	12.5	23.7	
t(8;21)	17.5	3.7	31.3	
Trisomies	14.4			
t(X;11)	13.2	0.0	31.3	
sAML2	12.1	9.9	14.2	
*TP53*–CK	5.3	2.9	7.6	
inv(3)	4.9	0.8	9.1	
*WT1*	4.0	0.0	18.4	

NR: Mean overall survival not reached at: “inv(16)”: 42 months; “No events”: 33 months and “*CEBPA* bZIP”: 31 months.

## Data Availability

The data presented in this study are available on request from the corresponding author.

## Supplementary Materials

The following supporting information can be downloaded at: https://www.mdpi.com/article/10.3390/cancers15020438/s1, Table S1: Description of the sequencing technology, NGS panel and NGS platform used by each reference laboratory; Table S2: Detailed gene panel composition; Table S3: Genomic groups and integrated risk score based on 16 molecular classes defined by Tazi et al., 2022 in the updated genomic classification of AML; Table S4: Third Cross–validation round results; Table S5: Molecular alterations pertaining to described functional categories. Table S6: Mutational frequency distribution according to moment disease, age and sex; Table S7: Hazard ratio for (A) molecular classes, (B) Risk score in global cohort, (C) Risk score in <65 years–old, (D) Risk score in ≥65 years–old; Figure S1: Third Cross–validation round results; Figure S2: Mutational frequency of PETHEMA consensus genes in the global cohort; Figure S3: Stability rates in diagnosis–relapse samples. Figure S4: Stability rates in diagnosis–refractoriness samples; Figure S5: Overall survival curves according to AML molecular classes; Figure S6: Overall survival curves of *TP53* mutated patients according to mono–allelic vs. multi–hit configurations.

## Appendix A

Institutions and clinicians participating in the PETHEMA epidemiologic registry of acute myeloid leukemia and acute promyelocytic leukemia.

Argentina (Grupo Argentino para el Tratamiento de la Leucemia Aguda—GATLA)—Hospital de Clínicas, Buenos Aires: F. Rojas; H. Longoni; Fundaleu, Buenos Aires: G. Milone, I. Fernández, Clínica Conciencia, Neuquén: R. Ramirez; Hospital Rossi, La Plata: C. Canepa, S. Saba, G. Balladares, Hospital General San Martin, Parana: G. Milone, C. Ventiurini, R. Mariano, P. Negri; Hospital Italiano de La Plata, La Plata: M. V. Prates, J. Milone; Hospital General San Martín, La Plata:P. Fazio, M. Gelemur; Hospital Clemente Alvarez, Rosario: G. Milone, S. Ciarlo, F. Bezares; Hospital de Córdoba, Córdoba: L. López, Hospital Privado de Córdoba, Córdoba: J. J.García; Instituto Privado Hematología, Paraná: P. Negri, M. Giunta, G. Milone; Hospital Teodoro Alvarez, Buenos Aires: M. Kruss; Hospital Tornú, Buenos Aires: D. Lafalse, G. Milone; Hospital Gobernador Centeno, La Pampa: E. Marquesoni, M. F. Casale; Hospital Italiano de Buenos Aires, Buenos Aires: A. Gimenez, E. B. Brulc, M. A. Perusini; Complejo Médico Policía Federal, La Plata: G. Milone, L. Palmer; Colombia (Asociación Colombian de Hematología y Oncología—ACHO)—Clínica La Estancia, Popayán: M. E. Correa; Fundación Valle del Lili, Cauca: F.J. Jaramillo, J. Rosales; Instituto FOSCAL, Bucaramanga: C. Sossa, J. C. Herrera; Hospital Pablo Tobón Uribe, Antioquia: M. Arango; Poland (Polish Adult Leukemia Group—PALG)—City Hospital Legnica, Baja Silesia: J. Holojda; IHIT Hematology and transfusiology institute, Warszawa: A. Golos, A. Ejduk; Wojewódzki Szpital Specjalistyczny w Olsztynie, Olsztyn: B. Ochrem; WIM (Military Institute of Medicine in Warsaw), Warszawa: G. Małgorzata; Poland Medical University of Warsaw Banacha, Warszawa:A. Waszczuk-Gajda, J. Drozd-Sokolowska, M. Czemerska, M. Paluszewska; Medical University School Gdansk, Gdansk: E. Zarzycka; Wojewódzki Szpital Specjalistyczny im. Św.. Jadwigi Śląskiej, Opole: A. Masternak; Hospital Brzozow, Brzozow: Dr. Hawrylecka; Medical University Lublin, Lublin: M. Podhoreka, K. Giannopoulos, T. Gromek; Medical University Bialystok, Bialystok: J. Oleksiuk; Silesian Medical University Katowice, Katowice:bA. Armatys, G. Helbig; Universitary Hospital Wroclaw, Wroclaw: M. Sobas; Poznan University of Medical Sciences, Pozna: A. Szczepaniak; Rydigier City Hospital Krakow, Krakow: E. Rzenno, M. Rodzaj; Collegium Medicum Jagiellonian University Krakow, Krakow: B. Piatkowska-Jakubas; City Hospital Rzeszów, Rzeszów: A. Skret; Medical University Lodz, Lodz: A. Pluta, M. Czemerska; Center of Oncology Kielce, Kielce: E. Barańska; Medical University of Warsaw, Warsaw: M. Paluszewska; Portugal—Hospital de Santa Maria-Lisboa, Lisboa:G. Vasconcelos, J. Brioso; IPOFG Lisboa, Lisboa: A. Nunes, I. Bogalho; Centro Hospitalar e Universitário de Coimbra, Coimbra: A. Espadana, M. Coucelo, S. Marini, J. Azevedo, A. I. Crisostomo, L. Ribeiro, V. Pereira; Centro Hospitalar de Lisboa Central E. P. E, Lisboa: A. Botelho; Instituto Português Oncologia do Porto Francisco Gentil, Porto: J. M. Mariz; Centro Hospitalar São João, Porto: J. E. Guimaraes, E. Aguiar; Centro Hospitalar do Porto E.P.E. Porto: J. Coutinho; Spain (Programa Español de Tratamiento de las Hemopatías Malignas, PETHEMA)—Complejo Hospitalario Universitario A Coruña, A Coruña: V. Noriega, L. García, C. Varela, G. Debén, M. R. González; Hospital Clínico Universitario de Santiago, A Coruña: M. Encinas, A. Bendaña, S. González, J.L. Bello, M. Albors; Hospital General de Albacete, Albacete: L. Algarra, J.R.Romero, J.S.Bermon, M.J. Varo; Hospital Vinalopó, Alicante: V. López, E. López; Hospital Virgen de los Lirios, Alcoy: C. Mora, C. Amorós; Hospital General Elche, Alicante: E. López, A. Romero; Hospital Torrevieja Salud, Alicante: A. Jaramillo, N. Valdez, I. Molina, A. Fernández, B. Sánchez; Hospital de la Marina Baixa Villajoyosa, Alicante: A. García; Hospital General de Elda, Alicante: V. Castaño, T. López, J. Bernabeu; Hospital de Denia-Marina Salud, Alicante: M.J. Sánchez; Hospital de la Vega Baja de Orihuela, Alicante: C. Fernández; Hospital General de Alicante, Alicante: C. Gil, C. Botella, P. Fernández, M. Pacheco, F. Tarín; J.J. Verdú; Complejo Hospitalario Torrecardenas, Almeria: M.J. García, A. Mellado, M.C. García, J. González; Hospital Central de Asturias, Asturias: T. Castillo, E. Colado, S. Alonso; Complejo Asistencial Ávila, Ávila: I. Recio, M. Cabezudo, J. Davila, M. J. Rodríguez, A. Barez, B. Díaz; Hospital Don Benito-Villanueva, Badajoz: J. Prieto; Institut Catala d’Oncologia L’Hospitalet, Barcelona: M. Arnan, C. Marín, M. Mansilla; Hospital de Cruces, Bizkaia: A. Balaberdi, M. E. Amutio, R. A. del Orbe, I. Ancin, J. C. Ruíz; Hospital Galdakao-Usansolo, Bizkaia: M. Olivalres, C. Gómez, I. gonzález, M. Celis, K. Atutxa, T. Carrascosa, T. Artola, M. Lizuain; Basurtuko Ospitalea, Bizkaia: J.I. Rodriguez, O. Arce, J. A. Márquez, J. Atuch, F. Marco de Lucas, Z. Díez, B. Dávila; Hospital Santos Reyes, Burgos: R. Cantalejo, M. Díaz; Hospital Universitario de Burgos, Burgos: J. Labrador, F. Serra, G. Hermida, F. J. Díaz, P. de Vicente, R. Álvarez: Hospital Santiago Apóstol, Burgos: C. Alonso, Hospital San Pedro de Alcántara, Cáceres: J. M. Bergua; Hospital Campo Arañuelo, Cáceres: N. Ugalde; Hospital Virgen del Puerto, Cáceres: E. Pardal; Hospital General Jerez de la Frontera, Cádiz: R. Saldaña, F. Rodríguez, E. Martín, L. Hermosín; Hospital Universitario Puerta del Mar, Cádiz: M. P. Garrastazul, I. Marchante, J. A. Raposo, F. J. Capote; Hospital U. Marqués de Valdecilla, Cantabria: M. Colorado, A. Batlle, L. Yañez, S. García, P. González, E. M. Ocio, M. Briz, A. Bermúdez, S. García; Consorcio Hospitalario Provincial de Castellón, Castellón: C. Jiménez, S. Beltrán; Hospital de Vinaroz: M. Montagud; Hospital Universitario de La Plana, Castellón: I. Castillo; Hospital General de Castellón, Castellon: R. García, A. Gascón, J. Clavel, A. Lancharro, L. Lnares; Hospital Santa Bárbara, Ciudad Real: M. M. Herráez, A. Milena; Hospital Virgen de Altagracia, Ciudad Real: M. J. Romero, Hospital General de Ciudad Real, Ciudad Real: B. Hernández, C. Calle, R. Benegas; Hospital Gutierrez Ortega de Valdepeñas, Ciudad Real: Dr. Bolívar; Hospital General La Mancha Centro, Ciudad Real: M. A. Pozas; Hospital Reina Sofia, Córdoba: J. Serrano, F. J. Dorado, J. Sánchez, M. C. Martínez; Hospital Virgen de la Luz, Cuenca: C. J. Cerveró, M. J. Busto; Hospitales HUVN-HC San Cecilio de Ganada, Granada: M. Bernal, E. López, L. Moratalla, Z. Mesa, M. Jurado, A. Romero, P. González; Complejo Hospitalario Universitario Granada, Granada: L. Moratalla, A. Romero, L. López; Hospital Universitario de Guadalajara, Guadalajara: M. Díaz, D. De Miguel, A. B. Santos, J. Arbeteta; Hospital Donostia, Donosti: E. Pérez, N. Caminos, N. Uresandi, N. Argoitiaituart, T. Artola, J. Swen, A. Uranga, I. Olazaba, M. Lizuain, E. Gainza, P. Romero; Hospital Juan Ramón Jimenez Huelva, Huelva: E. Gil, A. J. Palma, K. G. Gómez, M. Solé, J. N. Rodríguez; Hospital San Jorge, Huesca: I. M. Murillo, J. Marco, J. Serena, V. Marco; Hospital de Barbastro, Huesca: M. Perella, L. Costilla; Hospital General Ciudad de Jaen, Jaén: J. A. López, A. Baena, P. Almagro; Hospital San Pedro de Logroño, La Rioja: M. Hermosilla, A. Esteban, B. A. Campeny, M. J. Nájera, P. Herrra; Hospital Insular de Las Palmas, Las Palmas: R. Fernández, J. D. González, L. Torres; Hospital Dr. Negrín, Las Palmas: S. Jiménez; M. T. Gómez, C. Bilbao, C. Rodríguez; Hospital Doctor José Molina Orosa, Las Palmas: A. Hong, Y. Ramos de Laón, V. Afonso; Hospital Universitario de León, León: F. Ramos, M. Fuertes; Hospital Comarcal del Bierzo, León: E. de Cabo, C. Aguilera, M. Megido; Hospital Universitari Arnau de Vilanova de Lleida, Leida: T. García; Hospital Lucus Augusti, Lugo: E. Lavilla, M. Varela, S. Ferrero, M. J. Sánchez, L. López, J. Arias, L. Vizcaya; Hospital Infanta Sofía, Madrid: A. Roldán, A. Vilches, M. J. Penalva, J. Vázquez; Hospital Central de la Defensa Gómez Ulla, Madrid: M. T. Calderón, A. Matilla, C. Serí, M. J. Otero, N. García, E. Sandoval; Hospital de Fuenlabrada, Madrid: C. Franco, R. Flores, P. Bravo, A. López; Hospital Fundación Jiménez Díaz, Madrid: J. L. López, C. Blas, A. Díez, J. M. Alonso, C. Soto, A. Arenas; Hospital U. Príncipe de Asturias, Madrid: J. García, Y. Martín, P. S. Villafuerte, E. Magro; Hospital Puerta de Hierro, Madrid: G. Bautista; A. De Laiglesia; Hospital Gregorio Marañón, Madrid: G. Rodríguez, L. Solán, M. Chicano, P. Balsalobre, S. Monsalvo, P. Font, D. Carbonell, C. Martínez; Hospital U. La Paz, Madrid: K. Humala, A. E. Kerguelen, D. Hernández, M. Gasior, P. Gómez, I. Sánchez; Hospital Madrid Norte Sanchinarro, Madrid: S. Redondo, L. Llorente, M. Bengochea, J. Pérez; Hospital Sanitas Torrejón, Madrid: A. Sebrango, M. santero, A. Morales; Hospital La Princesa, Madrid: A. Figuera, P. Villafuerte, A. Alegre, E. Fernández; Hospital Ruber Internacional, Madrid: A. Alonso; Hospital 12 de Octubre, Madrid: M. P. Martínez, J. Martínez, M. T. Cedena, L. Moreno; MD Anderson Cancer Center, Madrid: A. De la Fuente; Hospital Sanitas La Zarzuela, Madrid: D. García; Hospital Universitario Quiron, Madrid: C. Chamorro, V. Pradillo, E. Martí, J. M. Sánchez, I. Delgado, A. Alonso; Hospital Rey Juan Carlos, Madrid: B. Rosado, A. Velasco, C. Miranda, G. Salvatierra, J. M. Alonso,, J. L. López; Hospital Infanta Leonor, Madrid: M. Foncillas, J. A. Hernández; Hospital Universitario de Getafe, Madrid: C. Escolano, L. García, I. Delgado; Hospital Clínico San Carlos, Madrid: C. Benabente, R. Martínez, M. Polo, E. Anguita; Hospital Universitario Severo Ochoa, Madrid: R. Riaza, G. Amores, M. J. Requena; Hospital Universitario Fundación Alcorcón, Madrid: F. Javier, L. Villaloón; Hospital Universitario Moncloa, Madrid: C. Aláez, V. Pradillo, S. Nistal, B. Navas; Hospital Universitario de Móstoles, Madrid: J. Sánchez, M. A. Andreu; Hospital Ramon y Cajal, Madrid: P. Herrera, J. López; Hospital U. Virgen de la Victoria, Málaga: M. García, M. J. Moreno, A. Fernández, M. P. Queipo; Hospital Quirónsalud Málaga, Málaga: A. Hernández; Hospital Regional de Málaga, Málaga: M. Barrios, A. Heiniger, A. Jiménez, A. Contento, F. López, M. Alcalá; Hospital Vithas Xanit Internacional, Málaga: S. Lorente, M. González, E. M. Morales, J. Gutierrez; Hospital Virgen del Castillo, Murcia: M. J. Serna, V. Beltrán; Hospital Santa Lucía de Cartagena, Murcia: M. Romera, M. Berenguer, A. Martínez, A. Tejedor; Hospital Morales Meseguer, Murcia: M. L. Amigo, F. Ortuño, L. García, A. Jerez, O. López; Hospital U. Virgen de la Arrixaca, Murcia: J. M. Moraleda, P. Rosique, J. Gómez, M. C. Garay; Hospital Los Arcos Mar Menor, Murcia: P. Cerezuela, C. Martínez, A. B. MArtínez, A. González; Hospital STª Mª del Rosell, Murcia: J. Ibáñez; Clínica San Miguel, Navarra: M. J. Alfaro; Complejo Hospitalario de Navarra, Navarra: M. Mateos, M. A. Goñi, M. A. Araiz, A. Gorosquieta, M. Zudaire, M. Viguria, A. Zabala, M. Alvarellos, I. Quispe, M. P. Sánchez, G. Hurtado, M. Pérez, Y. Burguete, N. Areizaga, T. Galicia; Clínica Universitaria de Navarra, Navarra: J. Rifón, A. Alfonso, F. Prósper, M. Marcos, L. E. Tamariz, V. Riego. A. Manubens, M. J. Larrayoz, M. J. Calasanz, A. Mañú, B. Paiva, I. Vázquez, L. Burgos; Complejo Hospitalario de Ourense (CHOU), Ourense: M. Pereiro, M. Rodríguez, M. C. Pastoriza, J. A. Mendez, J. L. Sastre, M. Iglesias, C. Ulibarrena, F. Campoy; Hospital Valdeorras, Ourense: D. Jaimes; Hospital Rio Carrión, Palencia: J. M. Alonso, B. Albarrán, J. Solano, A. Silvestre; Complexo Hospitalario Universitario de Vigo, Vigo: C. Albo, S. Suarez, C. Loureiro, I. Figueroa, M. Rodríguez, M. A. Fernández, A. Martínez, C. Poderós, J. Vazquez, L. Iglesias, A. Nieto, T. Torrado, A. M. Martínez; Hospital Provincial de Pontevedra, Pontevedra: M.L. Amador, P. Oubiña, E. Feijó, A. Dios, I. Loyola, R. Roreno; Hospital POVISA, Pontevedra: A. Simiele, L. Álvarez, V. Turcu; Hospital U. Salamanca, Salamanca: B. Vidriales, M. González, R. García, A. Avendaño, C. Chillón, E. Pérez, V. González; Hospital General La Palma, Santa Cruz de Tenerife: J. V. Govantes, S. Rubio, M. Tapia; Hospital General de Segovia, Segovia: C. Olivier, J. A. Queizán; Hospital U. Virgen Macarena, Sevilla: O. Pérez, J. A. Vera, C. Muñoz, A. rodriguez, N. González; Hospital U. Virgen del Rocio, Sevilla: J. A. Pérez, E. Soria, I.Espigado, J. Falantes, I. Montero, P. García, E. Rodríguez, E. Carrillo, T. Caballero, C. García; Hospital Virgen de Valme, Sevilla: C. Couto, I. Simón, M. Gómez; Hospital Virgen del Mirón de Soria, Soria: C. Aguilar; Hospital Universitario Canarias, Tenerife: B. J. González, S. Lakhwani, A. Bienert, B. González; Hospital Universitario Nuestra Señora de Candelaria, Tenrife: A. Cabello, A. Y. Oliva, H. González; Hospital Obispo Polanco, Teruel: N. González, Hospital de Alcañiz, Teruel: L. Sancho, M. Paricio, L. Perdiguer; Hospital General Nuestra Señora del Prado, Toledo: F. Solano, A. Lerma, M. D. Martínez; Hospital Virgen de la Salud de Toledo, Toledo: M. I. Gómez, A. Yeguas; Hospital U. La Fe, Valencia: P. Montesinos, E. Barragán, C. Sargas, R. Amigo, D. Martinez, B. Boluda, R. Rodríguez, E. Acuña, I. Cano; Hospital de Requena, Valencia: A. Escrivá, M. Pedreño; Hospital de Lluis Alcanyis de Xativa, Valencia: R. Renart; IVO (Instituto Valenciano de Oncología), Valencia: A. Navalón; Hospital de Sagunto, Valencia: I. Castillo, M. Orts; Hospital Dr. Peset, Valencia: M. J. Sayas, M. J. Fernández, M. L. Juan, E. Gómez, M. Gimeno, E. Donato, M. Cejalvo, J. Marco; Hospital Clínico Universitario, Valencia: M. Tormo, M. Calabuig, B. Navarro, I. Martin, E. Villamont, A. Miralles; Hospital de La Ribera, Valencia: R. Lluch; Hospital Casa de la Salud, Valencia: J. García; Hospital de Gandía, Valencia: M. Moragues, M. A. Ruiz; Hospital Arnau de Vilanova, Valencia: A. López, C. Benet, M. Valero; Hospital General de Valencia, Valencia: M. Linares, R. Collado, M. Orero, P. Ibañez, M. J. Lis, P. L. Pérez, M. Roig, M. López, A. V. Mena; Hospital Manises, Valencia: I. Picón, V. Cánovas, A. Palacios, E. Martí; Hospital Clínico de Valladolid, Valladolid: R. Cuello, J. Borrego, M. burgois; Hospital Rio Hortega, Valladolid: A. Cantalapiedra, O. Norberto, E. Angomas, B. Cidoncha; Hospital Universitario Araba, Victoria: L. Cuevas, D. Robles, A. Mendiazabal, I. Oiartzabal, J. M. Guinea de Castro; Hospital Virgen de la Concha, Zamora: C. Montes, M. Pérez, L. García; Hospital Royo Villanova, Zaragoza: V. Carrasco, A. Pérez, L. López, J. J. Moneva; Hospital Clínico U. Lozano Blesa, Zaragoza: M. Olave, E. Bonafonte, L. Mayor, G. Azaceta, L. Palomera; Hospital Ernest Lluch Martin, Zaragoza: M. Malo, M. J. Escobar; Hospital Quiron Salud Zaragoza, Zaragoza: J. M. Grasa; Hospital Miguel Servet, Zaragoza: B. De Rueda, A. Aulés, C. Salvador, V. Ansó, A. Iborra, P. Delagado, A. Rubio; Uruguay—Hospital de Clínicas, Montevideo: M. Stevenazzi, I. Alpire, V. Irigoin, L. Díaz, C. Guillermo, R. Guadagna, S. Grille, C. Oliver, M. Boada, V. Vales; Hospital Maciel, Montevideo: A. I. Prado; COMERO (Cooperativa Medica de Rocha), Rocha: A. P. De los Santos.
