# Supplementary material for: Molecular Landscape and Validation of New Genomic Classification in 2668 Adult AML Patients: Real Life Data from the PETHEMA Registry
Source: Cancers (Basel). 2023 Jan 10;15(2):438. doi: 10.3390/cancers15020438 (PMC9856266; doi:10.3390/cancers15020438)
Supplement: Supplementary file 1 [file cancers-15-00438-s001.zip › cancers-2081615-supplementary.pdf]

SUPPLEMENTARY MATERIAL:

Table S1. Description of the sequencing technology, NGS panel and NGS platform used by each reference laboratory.

Centers	Amplicon/Capture	Commercial or custom panel	Platform
HULF	Amplicon	Comercial: Oncomine Myeloid Reserach Assay (Thermo Fisher)	Ion S5 System (Thermo Fisher)
HUVR	Amplicon	Comercial: Oncomine Myeloid Reserach Assay (Thermo Fisher)	Ion GeneStudio S5 Prime (Thermo Fisher)
H12O	Amplicon	Custom: Ampliseq (Thermo Fisher)	Ion S5 System (Thermo Fisher)
HURS	Capture	Comercial: Myeloid Tumor Solution (SOPHiA GENETICS)	MiSeq System (Illumina)
HUDN	Capture	Comercial: Myeloid Tumor Solution (SOPHiA GENETICS)	MiSeq System (Illumina)
HUS	Capture	Custom: PanMyeloid (SOPHiA GENETICS)	MiSeq System (Illumina)
UNAV	Capture	Custom: PanMyeloid (SOPHiA GENETICS)	MiSeq System (Illumina)

HULF: Hospital Universitario La Fe, HUVR: Hospital Universitario Virgen del Rocío, H12O: Hospital Universitario 12 de Octubre, HURS: Hospital Universitario Reina Sofía, HUDN: Hospital Universitario de Gran Canaria Dr. Negrín, HUS: Hospital Universitario de Salamanca, UNAV: CIMA LAB Diagnostics.

Table S2. Detailed gene panel composition

Genes	HULF, HUVR	HURS, HUDN	H12O	HUS, UNAV
ABL1	4 to 9	4 to 9	-	-
ASXL1	All exons	10, 12, 13	All exons	12
BCOR	All exons	-	All exons	All exons
BRAF	2 to 4, 6, 8, 11, 15, 17 and 18	15	-	-
CALR	All exons	9	All exons	9
CBL	8, 9	8, 9	All exons	8, 9
CEBPA	All exons	All exons	All exons	All exons
CSF3R	14, 17	All exons	All exons	14 to 17
DNMT3A	11 to 23	All exons	All exons	All exons
ETV6	All exons	All exons	All exons	All exons
EZH2	All exons	All exons	All exons	All exons
FLT3	8, 11, 13 to 16, 20, 23, 24	13 to 15 and 20	All exons	14 to 16 and 20
GATA2	4, 5	-	-	2 to 6
HRAS	2, 3	2, 3	-	-
IDH1	4	4	All exons	4
IDH2	4	4	All exons	4
JAK2	12 to 15	All exons	All exons	12 to 15
KIT	1, 2, 8 to 11, 13, 17	2, 8 to 11, 13, 17 and 18	All exons	2, 8 to 11, 13, 14, 17
KRAS	2 to 6	2, 3	All exons	2 to 4
MPL	3, 4, 10, 12	10	All exons	3 to 6, 10, 12
NPM1	11	10, 11	All exons	10, 11
NRAS	2 to 4	2, 3	All exons	2 to 4
PTPN11	3, 12, 13	3, 7 to 13	-	3, 7, 13
RUNX1	All exons	All exons	All exons	All exons
SETBP1	4	4	All exons	4
SF3B1	14 to 21	10 to 16	All exons	11 to 16
SRSF2	1	1	All exons	1
STAG2	All exons	-	All exons	All exons
TET2	All exons	All exons	All exons	All exons
TP53	All exons	All exons	All exons	All exons
U2AF1	2, 6	2, 6	All exons	2, 6
WT1	7, 9	6 to 10	All exons	7, 9
ZRSR2	All exons	All exons	All exons	All exons

HULF: Hospital Universitario La Fe, HUVR: Hospital Universitario Virgen del Rocío, H12O: Hospital Universitario 12 de Octubre, HURS: Hospital Universitario Reina Sofía, HUDN: Hospital Universitario de Gran Canaria Dr. Negrín, HUS: Hospital Universitario de Salamanca, UNAV: CIMA LAB Diagnostics.

Table S3. Genomic groups and integrated risk score based on 16 molecular classes defined by Tazi *et al*., 2022 in the updated genomic classification of AML.

		**Risk groups**		
**Hierarchical model**	**Molecular classes**	**Favorable**	**Intermediate**	**Adverse**
**1. WHO 2016 Set 1**	*NPM1*	■		
	t(8;21)	■		
	inv(16)	■		
**2. *TP53* and/or complex karyotype (CK)**	*TP53* ±CK			■
**3. WHO 2016 Set 2**	*CEBPA* bi	■		
	t(11;x)		■	
	t(6;9)		■	
	inv(3)			■
**4. sAML1**: Single mutation in one of the following genes: *SRSF2, SF3B1, U2AF1, ASXL1, EZH2, RUNX1* and *SETBP1*	sAML1		■	
**5. sAML2:** Two or more mutations in sAML1 defining genes. *In this subset, *DNMT3A* and *TET2* account as a second hit	sAML2			■
**6. *WT1***	*WT1*		■	
**7. Trisomies:** Presence of ≥1 trisomies	Trisomies		■	
**8. *DNMT3A* + *IDH1/2***	*DNMT3A* + *IDH1/2*		■	
**9. Not class defining mutations**	mNOS		■	
**10. No events**	No events	■		

Table S4. Third Cross-validation round results

Variant ID	Gene	NM	Coding	Protein	Detected	Included	Error Rate	Mean VAF	SD
**Cross-Validation Round**									
**VAF≥5% variants**									
1	*JAK2*	(NM_004972)	c.1849G>T	p.(Val617Phe)	8	8	0,0%	80,9%	2,0%
2	*TP53*	(NM_000546.5)	c.833C>T	p.(Pro278Leu)	8	8	0,0%	80,3%	2,5%
3	*RUNX1*	(NM_001754.4)	c.592G>A	p.(Asp198Asn)	8	8	0,0%	63,2%	2,0%
4	*SRSF2*	(NM_003016.4)	c.284C>A	p.(Pro95His)	8	8	0,0%	49,6%	3,8%
5	*IDH2*	(NM_002168.3)	c.419G>A	p.(Arg140Gln)	8	8	0,0%	46,8%	1,8%
6	*DNMT3A*	(NM_022552.4)	c.2644C>T	p.(Arg882Cys)	8	8	0,0%	44,1%	1,5%
7	*ASXL1*	(NM_015338)	c.1934dup	p.(Gly646Trpfs*12)	5	8	37.5%	43,5%	4,5%
8	*IDH1*	(NM_005896.3)	c.395G>A	p.(Arg132His)	8	8	0,0%	41,3%	1,9%
9	*NPM1*	(NM_002520.6)	c.860_863dup	p.(Trp288Cysfs*12)	8	8	0,0%	41,1%	7,9%
10	*NPM1*	(NM_002520.6)	c.860_863dup	p.(Trp288Cysfs*12)	8	8	0,0%	37,3%	4,4%
11	*TET2*	(NM_001127208.2)	c.3814G>C	p.(Ala1272Pro)	7	8	12,5%	31,3%	5,8%
12	*FLT3*	(NM_004119.2)	c.2027A>C	p.(Asn676Thr)	6	8	25,0%	22,8%	1,9%
13	*CSF3R*	(NM_156039.3)	c.2308C>T	p.(Gln770*)	6	8	12,5%	15,7%	0,8%
14	*FLT3*	(NM_004119.2)	c.2504A>T	p.(Asp835Val)	8	8	0,0%	14,1%	0,9%
15	*FLT3*	(NM_004119.2)	c.1770_1796dup	p.(Tyr591_Tyr599dup)	7	8	12,5%	13,2%	3,5%
16	*KRAS*	(NM_033360.3)	c.182A>C	p.(Gln61Pro)	8	8	0,0%	13,1%	2,4%
17	*NRAS*	(NM_002524.4)	c.35G>A	p.(Gly12Asp)	8	8	0,0%	10,3%	1,1%
18	*PTPN11*	(NM_002834)	c.218C>T	p.(Thr73Ile)	7	8	12,5%	7,5%	1,0%
19	*SETBP1*	(NM_015559.2)	c.2602G>A	p.(Asp868Asn)	8	8	0,0%	7,3%	0,5%
20	*CSF3R*	(NM_156039.3)	c.2296C>T	p.(Gln766*)	6	8	12,5%	5,6%	0,7%
21	*NRAS*	(NM_002524.4)	c.35G>A	p.(Gly12Asp)	8	8	0,0%	5,0%	0,9%
**VAF<5% variants**									
22	*FLT3*	(NM_004119.2)	c.2522A>T	p.(Asn841Ile)	7	8	12,5%	3,7%	0,7%
23	*TP53*	(NM_000546.5)	c.742C>G	p.(Arg248Gly)	6	8	25,0%	2,4%	0,5%
24	*FLT3*	(NM_004119.2)	c.2505T>A	p.(Asp835Glu)	4	8	50,0%	1,6%	0,2%
25	*KRAS*	(NM_033360.3)	c.38G>A	p.(Gly13Asp)	3	8	62,5%	1,9%	0,8%
26	*JAK2*	(NM_004972)	c.1849G>T	p.(Val617Phe)	1	8	87,5%	1,4%	N/A
27	*NRAS*	(NM_002524.4)	c.35G>A	p.(Gly12Asp)	4	8	50,0%	1,4%	0,2%
28	*PTPN11*	(NM_002834)	c.1505C>T	p.(Ser502Leu)	3	8	62,5%	1,1%	0,1%
29	*PTPN11*	(NM_002834)	c.213T>G	p.(Phe71Leu)	1	8	87,5%	1,0%	N/A
30	*FLT3*	(NM_004119.2)	c.1812_1813ins30	p.(Glu604_605ins10)	4	8	50,0%	1,0%	0,2%
31	*PTPN11*	(NM_002834)	c.227A>G	p.(Glu76Gly)	1	8	87,5%	1,0%	N/A
32	*FLT3*	(NM_004119.2)	c.1740_1788dup	p.(Tyr597Glyfs*18)	5	8	37,5%	0,8%	0,4%

Detected: number of centers which have detected the variant; Included: number of centers which include variant region in its next-generation sequencing assay; Error Rate: percentage of centers which failed variant detection; VAF: variant allele frequency; SD: VAF standard deviation; NA: not applicable: variants only were detected by one center.

Table S5. Molecular alterations pertaining to described functional categories.

Functional categories	Molecular alterations
Transcription factor (TF) fusions	-
NPM1	*NPM1*
Tumor suppresors	*TP53* and *WT1*
DNA methylation	*DNMT3A* , *IDH1* , *IDH2* and *TET2*
Activating signaling	*ABL1* , *BRAF* , *FLT3* , *HRAS* , *JAK2* , *KIT* , *KRAS* , *NRAS* and *PTPN11*
Myeloid TF	*CEBPA* , *ETV6* , *GATA2* and *RUNX1*
Chromatin modifiers	*ASXL1* and *EZH2*
Spliceosome	*SF3B1* , *SRSF2* and *U2AF1*
Cohesin	-

Table S6. Mutational frequency distribution according to moment disease, age and sex. Bold format means statistically significant values.

**Genes**	**A) Disease status**				**B) Age**			**C) Sex**		
	**Diagnosis**	**Relapse**	**Refractoriness**	***P***	**≤65 years**	**>65 years**	***P***	**Male**	**Female**	***P***
	**%**	**%**	**%**		**%**	**%**		**%**	**%**	
ABL1	0.3%	0.9%	1.2%		0.3%	0.3%		0.2%	0.4%	
ASXL1	14.7%	16.3%	14.6%		**10.2%**	**18.4%**	**<0.001**	**17.7%**	**10.8%**	**<0.001**
BRAF	0.6%	0.0%	1.2%		0.6%	0.5%		0.4%	0.8%	
CALR	1.6%	3.6%	1.2%		1.4%	1.8%		1.7%	1.5%	
CBL	3.7%	2.8%	2.9%		3.5%	3.9%		**4.5%**	**2.8%**	**0.025**
CEBPA	6.2%	6.8%	3.5%		6.4%	6.1%		6.9%	5.4%	
CSF3R	2.8%	3.6%	2.3%		2.9%	2.7%		2.6%	3.0%	
DNMT3A	**23.8%**	**31.5%**	**21.6%**	**0.018**	**25.9%**	**22.1%**	**0.032**	**20.6%**	**27.9%**	**<0.001**
ETV6	3.1%	4.8%	2.3%		2.5%	3.6%		3.7%	2.4%	
EZH2	5.5%	5.2%	5.8%		**3.0%**	**7.5%**	**<0.001**	**7.0%**	**3.5%**	**<0.001**
FLT3	24.9%	24.3%	22.2%		**29.4%**	**21.3%**	**<0.001**	**22.2%**	**28.3%**	**<0.001**
GATA2	2.8%	4.4%	4.1%		3.4%	2.2%		3.0%	2.4%	
IDH1	**9.7%**	**15.1%**	**8.2%**	**0.017**	8.8%	10.4%		9.2%	10.4%	
IDH2	14.7%	15.5%	15.2%		**12.4%**	**16.5%**	**<0.001**	15.5%	13.6%	
JAK2	5.4%	3.2%	5.8%		**3.6%**	**6.9%**	**<0.001**	**6.4%**	**4.1%**	**0.01**
KIT	3.4%	4.4%	1.8%		4.2%	2.8%		3.3%	3.5%	
KRAS	**8.2%**	**3.6%**	**4.1%**	**0.007**	7.6%	8.6%		8.6%	7.6%	
MPL	1.8%	1.6%	1.2%		2.0%	1.7%		1.4%	2.4%	
NPM1	**23.3%**	**23.1%**	**9.4%**	**<0.001**	**29.2%**	**18.5%**	**<0.001**	**19.8%**	**27.7%**	**<0.001**
NRAS	**16.2%**	**8.0%**	**17.5%**	**<0.001**	**19.1%**	**13.9%**	**<0.001**	15.9%	16.5%	
PTPN11	5.8%	4.8%	5.3%		**7.3%**	**4.5%**	**<0.001**	5.8%	5.7%	
RUNX1	**18.2%**	**23.5%**	**23.4%**	**0.037**	**13.4%**	**21.9%**	**<0.001**	**21.1%**	**14.2%**	**<0.001**
SETBP1	3.0%	4.8%	4.7%		2.3%	3.5%		3.3%	2.6%	
SF3B1	5.7%	7.2%	9.5%		**4.5%**	**6.7%**	**0.028**	5.8%	5.7%	
SRSF2	16.1%	12.7%	16.4%		**8.1%**	**22.5%**	**<0.001**	**20.9%**	**9.8%**	**<0.001**
TET2	22.0%	19.1%	19.9%		**14.3%**	**28.2%**	**<0.001**	**23.8%**	**19.7%**	**0.014**
TP53	**17.9%**	**11.2%**	**20.5%**	**0.016**	**12.7%**	**22.0%**	**<0.001**	17.2%	18.8%	
U2AF1	6.5%	3.2%	8.2%		**4.9%**	**7.7%**	**<0.001**	**8.9%**	**3.2%**	**<0.001**
WT1	**4.8%**	**11.6%**	**10.5%**	**<0.001**	**6.3%**	**3.5%**	**<0.001**	4.1%	5.6%	

Table S7. Hazard ratio for A) molecular classes, B) Risk score in global cohort, C) Risk score in <65 years-old, D) Risk score in ≥65 years-old.

	Hazard ratio	(95% CI)		*P*
		Lower IC	Upper IC	
**A) Molecular classes**				
NPM1	Reference			-
CEBPA bZIP	0,3	0,0	2,0	0.201
inv(3)	3,9	2,1	7,2	<0.001
sAML1	1,4	1,0	2,1	0.051
sAML2	2,1	1,6	2,7	<0.001
WT1	2,3	1,1	5,0	<0.05
Trisomies	0,9	0,2	3,6	0.855
DNMT3A/IDH1-2	1,4	0,7	3,1	0.348
Not class defining mutations	0,9	0,6	1,4	0.753
No events	0,5	0,1	2,2	0.402
t(X;11)	1,8	0,9	3,4	0.093
inv(16)	0,9	0,5	1,8	0.758
TP53-CK	3,5	2,6	4,6	<0.001
t(8;21)	1,3	0,6	2,7	0.465
**B) Risk score in global cohort**				
Favorable	Reference			-
Intermediate	1,5	1,1	2,0	<0.01
Adverse	2,7	2,1	3,5	<0.001
**C) Risk classification in <65 years**				
Favorable	Reference			-
Intermediate	2,6	1,5	4,4	<0.001
Adverse	3,5	2,1	5,8	<0.001
**D) Risk classification in ≥65 years**				
Favorable	Reference			-
Intermediate	1,0	0,7	1,4	0.864
Adverse	1,9	1,4	2,5	<0.001

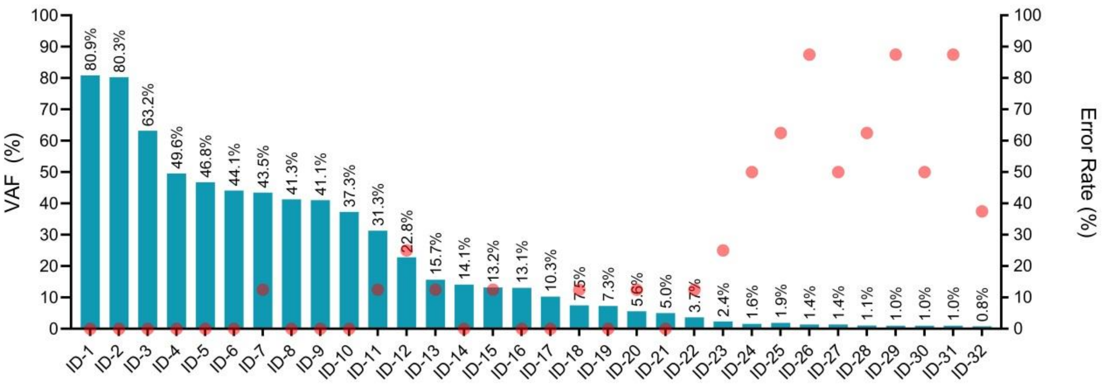

Figure S1. Third Cross-validation round results. Blue bars represent the allele frequency for the detected variants in the third cross-validation round. The red dots show the error rate in variant detection. The X-axis indicates the variant ID of table S2 for variant identification.

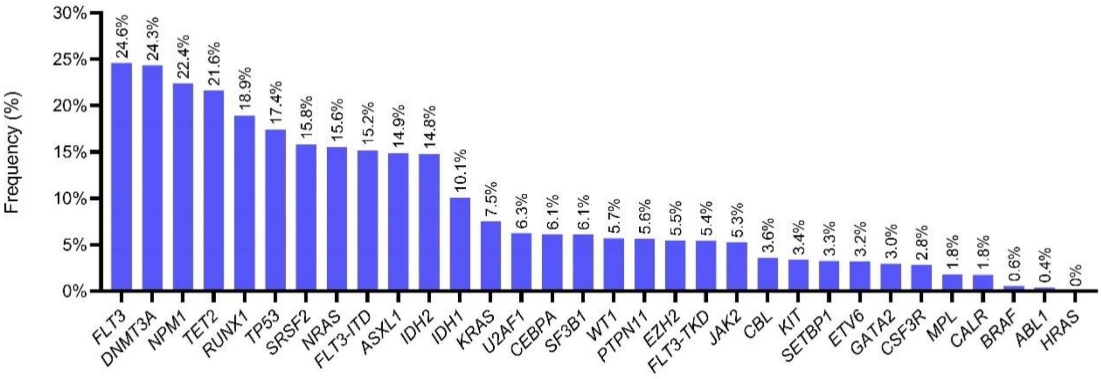

Figure S2. Mutational frequency of PETHEMA consensus genes in the global cohort (N=2856 samples).

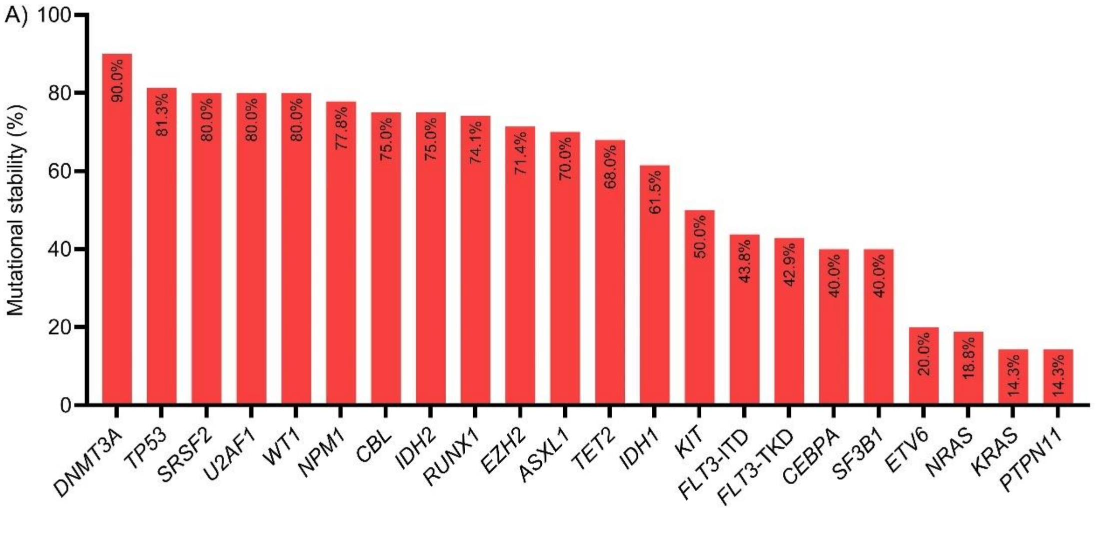

Figure S3. Stability rates in diagnosis-relapse samples (n=97 patients)

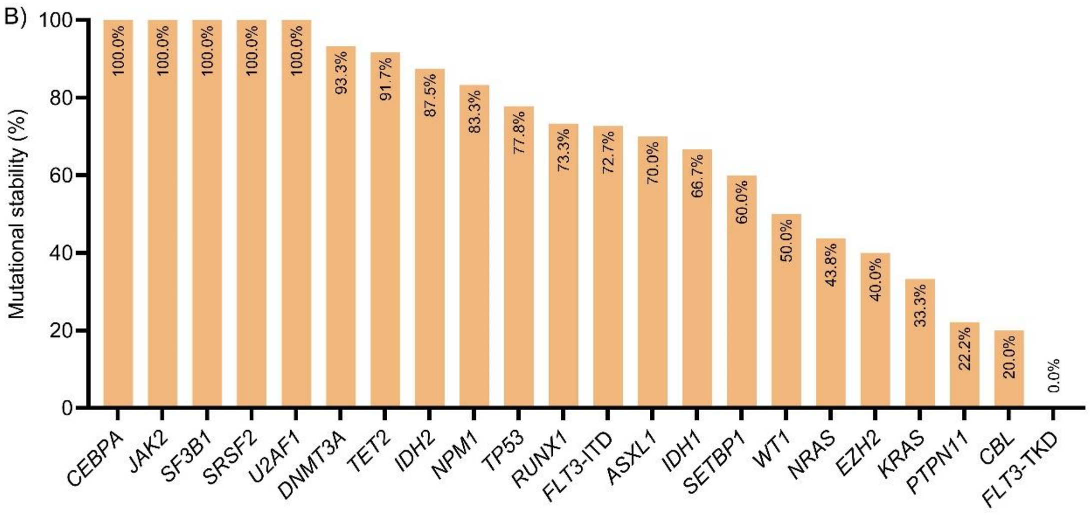

Figure S4. Stability rates in diagnosis-refractoriness samples (n=59 patients)

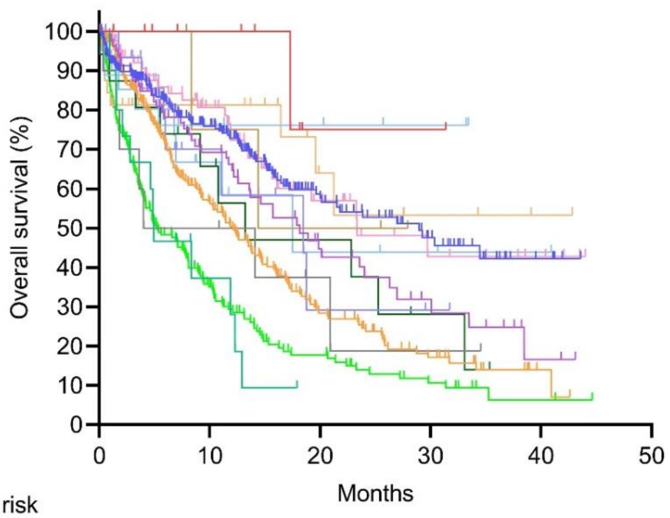

Number at risk					
NPM1	228	132	54	24	4
CEBPA bZIP	11	6	1	1	-
inv(3)	15	4	-	-	-
sAML1	83	46	21	9	2
sAML2	244	108	38	17	2
WT1	10	5	2	1	-
Trisomies	5	3	2	-	-
DNMT3A/IDH1-2	17	6	2	1	-
mNOS	79	41	17	8	4
No events	9	5	4	2	-
t(X;11)	17	8	5	2	-
inv(16)	32	14	6	3	1
TP53/CK	183	50	20	9	2
t(8;21)	21	8	3	2	1

Figure S5. Overall survival curves according to AML molecular classes.

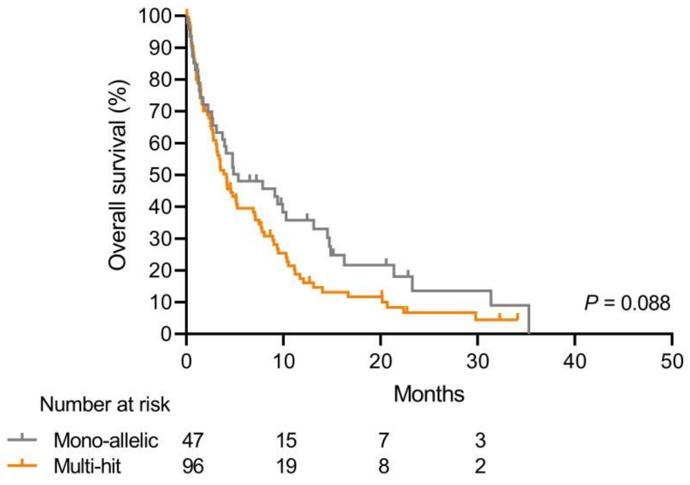

Figure S6: Overall survival curves of *TP53* mutated patients according to mono-allelic vs. multi-hit configurations.

Genetic analyses

NGS studies were performed in central laboratories according to specific protocols and sequencing platforms. Cytogenetic and molecular data were anonymously recruited by the diagnostic platform.
